# The impact of preference-based, person-centered care on regulatory outcomes

**DOI:** 10.1093/geroni/igaa057.3067

**Published:** 2020-12-16

**Authors:** Xiao Qiu, Katherine Abbott, John Bowblis, Kimberly Van Haitsma

**Affiliations:** 1 Miami University, Oxford, Ohio, United States; 2 Penn State University, University Park, Pennsylvania, United States

## Abstract

The Preferences for Everyday Living Inventory (PELI) was mandated as a pay for performance indicator by the Ohio Department of Medicaid in 2015. This study explored the impacts of PELI implementation on regulatory outcomes in 2017.

The level of PELI implementation from n=551 Ohio nursing home providers between 2015 and 2017 were linked with Centers for Medicare and Medicaid Services Nursing Home Compare data. Fixed effects panel regression analyses assessed the effects of time-varying PELI implementation on 2015-2017 regulatory outcomes that could be correlated with quality of life including fines, substantiated complaints, health scores, deficiency counts and deficiency scores.

Results show a significant increase in substantiated complaints among providers that were slow adopters of the PELI. Overall, the extent of PELI implementation was not associated with regulatory outcomes.

The use of the PELI may not impact substantiated complaints suggesting further research is needed to identify person-centered outcomes of interest. Part of a symposium sponsored by the Research in Quality of Care Interest Group.

